# Supplementary material for: Altered thalamocortical structural connectivity in persons with schizophrenia and healthy siblings
Source: Neuroimage Clin. 2020 Jul 31;28:102370. doi: 10.1016/j.nicl.2020.102370 (PMC7451425; doi:10.1016/j.nicl.2020.102370)
Supplement: Supplementary data 1 [file mmc1.docx]

**Supplementary Methods**

**Participants**

Within the schizophrenia group, 16 individuals had a diagnosis of schizophrenia and three were diagnosed with schizoaffective disorder. The remaining three had been diagnostically characterized using the Comprehensive Assessment of Symptoms and History interview (1) by another study group at an outpatient psychiatric facility and met diagnostic criteria for schizophrenia or schizoaffective disorder. Within the healthy sibling group, 15 had a sibling with schizophrenia, four had a sibling with schizoaffective disorder, and one had a sibling with Psychosis NOS at the time of assessment.

**Diffusion-weighted imaging**

*Head movements.* Head movements are reported in **Table S5**. Participant head movements were quantified by calculating the Root Mean Square deviation between the affine transformation matrices produced by the *eddy_correct* procedure using the *rmsdiff* utility in FSL. Average displacements and head rotations from both phase-encoding directions were examined using one-way ANOVAs. No group differences were observed for absolute total displacements (*F*(2,83) = 2.17, *p* = 0.12; *F*(2,83) = 2.91, *p* = 0.06), relative total displacements (*F*(2,83) = 1.71, *p* = 0.19; *F*(2,83) = 1.59, *p* = 0.21), or head rotations in any dimension (see **Table S5** for p-values).

**Statistical analysis**

*Group differences in ROIs.* First, we examined if there were group differences in volumes of thalamus and cortical ROIs. A careful examination of the Q-Q plots revealed that total volumes of thalamus and cortical ROIs were all normally distributed. Box’s M test revealed that the assumption of homoscedasticity was met (*M* = 286.04, *p* = .62). To protect against inflated Type I error due to multiple comparisons (2), a MANCOVA was conducted to examine if there were overall group differences across the volumes of the six ROI and the thalamus. Diagnostic group was included as a between-subject variable, and age, sex, and whole brain volume as covariates. Hemisphere was included as a within-subject variable.

Next, we examined potential group differences in voxels included in the thalamus mask. To this end, individual thalamus masks were normalized to MNI space and then binarized. These individual masks were then summed within each group to create three group thalamus count maps, where each voxel represents the number of individuals for whom that voxel was included in their thalamus mask. The group inclusion mask created for the voxel-wise analysis (that excluded voxels from which very few streamlines terminated in cortex) was than applied to the three group thalamus count maps. A Fisher’s exact test was conducted on the group maps at each voxel to calculate the exact probability of observing the data, and the resulting *p* value at each voxel comprised a thalamic *p*-value map. This map was thresholded at p<0.005 with a cluster extent threshold ≥ 10 voxels), following published guidelines in this journal (3, 4).

**Supplementary Results**

**Group differences in ROIs**

Total volumes of thalamus and cortical ROI are presented in **Table S2.** Across all ROIs, the MANCOVA test revealed significant effects of hemisphere (*F*(7.77) = 41.83, *p* < .001; Wilk’s Λ = 0.21, partial *η^2^* = 0.79), age (*F*(7,74) = 4.90, *p* < .001; Wilk’s Λ = 0.68, partial *η^2^* = 0.32), and whole brain volume (*F*(7,74) = 69.22, *p* < .001; Wilk’s Λ = 0.13, partial *η^2^* = 0.87). There were no significant effects of group (*F*(14,148) = 0.91, *p* = .55; Wilk’s Λ = 0.85, partial *η^2^* = 0.08) and sex (*F*(7,74) = 1.55, *p* = .17; Wilk’s Λ = 0.87, partial *η^2^* = 0. 13). Moreover, there were no significant differences in voxel composition of thalamic masks, suggesting no systematic group differences in the creation of thalamus masks.

**Cross-thalamic cortical connectivity including sex as a covariate**

A MANCOVA was conducted to examine if there were overall group differences across the six percent connectivity variables between cortical ROI and the thalamus, including diagnostic group as a between-subject variable, hemisphere as a within-subject variable, and age and sex as covariates. Across all six cortical ROIs, there was a trend-level main effect of group (*F*(12,152) = 1.65, *p* = .08; Wilk's Λ = 0.78, partial *η^2^* = 0.12) and a significant main effect of hemisphere (*F*(6,78) = 8.91, *p* < .001; Wilk’s Λ = 0.59, partial *η^2^* = 0.41). There were no significant effects of age (*F*(6,76) = 0.82, *p* = .56; Wilk's Λ = 0.94, partial *η^2^* = 0.06) and sex (*F*(6,76) = 1.09, *p* = .37; Wilk's Λ = 0.92, partial *η^2^* = 0.08). Although the p-value of the group effect dropped to just below statistical significance when adding sex as a factor in the model, we do not think that sex confounded the group effect in thalamo-cortical connectivity as reported in the main text. Instead, the drop in statistical significance is more likely due to reduced statistical power after including another variable in the model, for the following reasons. First, the effect of sex was not significant and the sex distribution is not imbalanced across groups (*χ^2^* = 4.79, *p* = .09). Further supporting this conclusion was the fact that the effect size of group differences barely changed (partial *η^2^* = 0.13 to 0.12). To formally test this, we converted the *η^2^* to *r* and performed a Steiger's *z* test (5). The change in effect size was not statistically significant (*z* = 0.98, *p* = .33). Therefore, we followed up the trend-level main effect of group with univariate tests.

For connections between thalamus and prefrontal cortex, there was a significant main effect of group (*F*(2,81) = 5.02, *p* = .009, partial *η^2^* = 0.11). Post Hoc analyses after Sidak adjustment revealed that, compared to HC, both SIB (*p* = .03) and SZ (*p* = .04) had significantly lower thalamo-prefrontal connectivity. SIB and SZ did not differ from each other (*p* = .99). There was no significant effect of hemisphere (*F*(1,83) = 0.78, *p* = .38, partial *η^2^* = 0.01), nor any group by hemisphere interaction (*F*(2,83) = 1.29, *p* = .28, partial *η^2^* = 0.03). For connections between thalamus and motor cortex, there was also a significant effect of group

(*F*(2,81) = 3.95, *p* = .02, partial *η^2^* = 0.09). Post-hoc tests revealed significantly higher connectivity in the SZ than HC group (*p* = .02), while SIB did not differ significantly from either

HC (*p* = 0.96) or SZ (*p* = .11). There was also a significant main effect of hemisphere (*F*(1,83) = 22.90, *p* < .001, partial *η^2^* = 0.22), with thalamo-motor connectivity being higher in the left hemisphere, but no significant group-by-hemisphere interaction effect (*F*(2,83) = 0.31, *p* = .73, partial *η^2^* = 0.01).

There was no group difference in percent connectivity between thalamus and any other cortical ROI (Somatosensory: *F*(2,81) = 2.71, *p* = .07, partial *η^2^* = 0.06; Posterior Parietal: *F*(2,81) = 2.71, *p* = .07, partial *η^2^* = 0.06; Temporal: *F*(2,81) = 0.15, *p* = .86, partial *η^2^* = 0.004; Occipital: *F*(2,81) = 1.50, *p* = .23, partial *η^2^* = 0.04). There was, however, a significant effect of hemisphere on thalamo-posterior parietal (*F*(1,83) = 27.73, *p* < .001, partial *η^2^* = 0.25) and thalamo-temporal (*F*(1,83) = 11.07, *p* = .001, partial *η^2^* = 0.12) connectivity. Thalamo-posterior parietal connectivity was higher in the right hemisphere, while thalamo-temporal connectivity was higher in the left hemisphere. There was no hemisphere difference in percent connectivity between thalamus and somatosensory cortex (*F*(1,83) = 2.60, *p* = .11, partial *η^2^* = 0.03) and between thalamus and occipital cortex (*F*(1,83) = 1.99, *p* = .16, partial *η^2^* = 0.02). Lastly, there was no significant group × hemisphere interaction effect on connectivity between thalamus and either somatosensory (*F*(2,83) = 2.88, *p* = .06, partial *η^2^* = 0.06), posterior parietal (*F*(2,83) = 0.29, *p* = .75, partial *η^2^* = 0.01), temporal (*F*(2,83) = 2.37, *p* = .10, partial *η^2^* = 0.05), or occipital (*F*(2,83) = 1.07, *p* = .35, partial *η^2^* = 0.03) cortex.

Lastly, there was no effect of age on connectivity between thalamus and any of the cortical ROIs (.08 ≤ *p* ≤ .87). There was a significant effect of sex on thalamo-temporal connectivity (*p* = .03), such that the connectivity was higher in males than in females across hemispheres. There was no effect of sex on connectivity between thalamus and the other cortical ROIs (.10 ≤ *p* ≤ .80).

**Table S1.** Parcellation structures included in cortical regions of interest (ROIs).

|  |  | Freesurfer Label | |
| --- | --- | --- | --- |
| Cortical ROI | Parcellation Structure | Left | Right |
| Prefrontal Cortex | Caudal Anterior (Frontal) | 1002 | 2002 |
|  | Caudal Middle Frontal | 1003 | 2003 |
|  | Lateral Orbitofrontal | 1012 | 2012 |
|  | Medial Orbitofrontal | 1014 | 2014 |
|  | Pars Opercularis | 1018 | 2018 |
|  | Pars Orbitalis | 1019 | 2019 |
|  | Pars Triangularis | 1020 | 2020 |
|  | Rostral Anterior Cingulate | 1026 | 2026 |
|  | Rostral Middle Frontal | 1027 | 2027 |
|  | Superior Frontal | 1028 | 2028 |
|  | Frontal Pole | 1032 | 2032 |
| Motor Cortex | Paracentral | 1017 | 2017 |
|  | Precentral | 1024 | 2024 |
| Somatosensory Cortex | Postcentral | 1022 | 2022 |
| Posterior Parietal Cortex | Inferior Parietal | 1008 | 2008 |
|  | Isthmus Cingulate | 1010 | 2010 |
|  | Posterior Cingulate | 1023 | 2023 |
|  | Precuneus | 1025 | 2025 |
|  | Superior Parietal | 1029 | 2029 |
|  | Supramarginal | 1031 | 2031 |
| Temporal Cortex | Banks of the Superior Temporal Sulcus | 1001 | 2001 |
|  | Entorhinal | 1006 | 2006 |
|  | Fusiform | 1007 | 2007 |
|  | Inferior Temporal | 1009 | 2009 |
|  | Middle Temporal | 1015 | 2015 |
|  | Parahippocampal | 1016 | 2016 |
|  | Superior Temporal | 1030 | 2030 |
|  | Temporal Pole | 1033 | 2033 |
|  | Transverse Temporal | 1034 | 2034 |
| Occipital Cortex | Cuneus | 1005 | 2005 |
|  | Lateral Occipital | 1011 | 2011 |
|  | Lingual | 1013 | 2013 |
|  | Pericalcarine | 1021 | 2021 |

**Table S2.** Volume (mm^3^) of cortical ROI and thalamus.

|  |  | SZ (*N* = 22) | SIB (*N* = 20) | HC (*N* = 44) |
| --- | --- | --- | --- | --- |
| ROI mask | Hemisphere | Mean (SD) | Mean (SD) | Mean (SD) |
| Prefrontal Cortex | Left | 69645.95 (7888.20) | 74453.50 (9557.63) | 75306.41 (8182.95) |
|  | Right | 70460.86 (8808.35) | 75935.05 (9992.73) | 76510.98 (8413.74) |
| Motor Cortex | Left | 16854.82 (2374.30) | 17405.70 (1775.24) | 17585.84 (1721.87) |
|  | Right | 17091.32 (2542.19) | 17968.65 (2147.37) | 18119.68 (2060.39) |
| Somatosensory Cortex | Left | 9755.41 (1428.69) | 10291.20 (1518.60) | 10006.41 (1230.76) |
|  | Right | 9192.27 (1524.76) | 9774.10 (1435.01) | 9468.32 (1372.00) |
| Posterior Parietal Cortex | Left | 51273.77 (6857.94) | 55973.50 (7921.42) | 54759.14 (6245.77) |
|  | Right | 52142.77 (6785.53) | 56487.80 (7856.04) | 55984.09 (6079.45) |
| Temporal Cortex | Left | 52348.59 (6088.48) | 57119.80 (8097.69) | 56606.84 (5582.12) |
|  | Right | 51325.68 (6301.18) | 56367.10 (7773.48) | 55662.93 (5350.92) |
| Occipital Cortex | Left | 23323.05 (2967.79) | 24588.75 (3112.77) | 24254.00 (3268.96) |
|  | Right | 23393.27 (3223.77) | 25119.70 (3136.38) | 24415.98 (3093.45) |
| Thalamus | Left | 7827.23 (978.68) | 8073.46 (898.42) | 7726.42 (915.21) |
|  | Right | 6965.26 (869.93) | 7311.78 (685.80) | 7049.22 (697.73) |

Notes: HC, healthy control participants; SIB, healthy siblings; SZ, persons with schizophrenia.

**Table S3.** Thalamocortical connectivity voxel-wise analysis results (including age as a covariate).

|  |  | Cluster-level | | |  | Peak-level | | | MNI Coordinates | | |
| --- | --- | --- | --- | --- | --- | --- | --- | --- | --- | --- | --- |
| Cortical Target | Hemisphere | *p*_FWE-corr_ | Cluster Size (voxels) | *p*_uncorr_ |  | *p*_FWE-corr_ | *F* | *p*_uncorr_ | mm | mm | mm |
| Motor | Left | .027 | 185 | .062 |  | .072 | 8.28 | .001 | -24 | -24 | 12 |
|  |  |  |  |  |  | .159 | 7.04 | .002 | -10 | -30 | 6 |
|  |  |  |  |  |  | .215 | 6.55 | .002 | -8 | -20 | 10 |
|  | Right | .108 | 51 | .187 |  | .189 | 7.27 | .001 | 16 | -14 | 4 |
| Occipital | Left | .274 | 11 | .602 |  | .104 | 8.02 | .001 | -4 | -20 | 16 |
|  | Right | no significant clusters | | | | | | | | | |
| Posterior Parietal | Left | no significant clusters | | | | | | | | | |
|  | Right |  |  |  |  |  |  |  |  |  |  |
| Prefrontal | Left | .137 | 44 | .266 |  | .160 | 7.39 | .001 | -18 | -26 | 6 |
|  |  |  |  |  |  | .239 | 6.72 | .002 | -6 | -32 | 2 |
|  | Right | no significant clusters | | | | | | | | | |
| Somatosensory | Left | no significant clusters | | | | | | | | | |
|  | Right | .210 | 18 | .545 |  | .017 | 10.38 | <.001 | 28 | -22 | -6 |
|  |  | .234 | 13 | .613 |  | .246 | 6.30 | .003 | 2 | -4 | -6 |
|  |  |  |  |  |  | .284 | 6.05 | .004 | 6 | -6 | 2 |
| Temporal | Left | .098 | 62 | .179 |  | .052 | 9.20 | <.001 | -24 | -24 | -4 |
|  |  |  |  |  |  | .115 | 7.97 | .001 | -28 | -32 | 2 |
|  |  | .197 | 26 | .382 |  | .188 | 7.18 | .001 | -8 | -24 | 8 |
|  | Right | no significant clusters | | | | | | | | | |

Notes: results were significant at voxel-wise uncorrected *p* < .005 and cluster extent threshold ≥ 10 voxels.

FWE-corr, Family-wise error corrected; uncorr, uncorrected.

**Table S4.** Thalamocortical connectivity voxel-wise analysis pairwise comparison results.

|  |  | Cluster-level | | |  | Peak-level | | | MNI Coordinates | | |
| --- | --- | --- | --- | --- | --- | --- | --- | --- | --- | --- | --- |
| Cortical Target | Contrast | *p*_FWE-corr_ | Cluster Size (voxels) | *p_uncorr_* |  | *p*_FWE-corr_ | *T* | *p*_uncorr_ | mm | mm | mm |
| Left Motor | HC < SIB | .011 | 386 | .034 |  | .013 | 4.00 | <.001 | -24 | -32 | 10 |
|  |  |  |  |  |  | .054 | 3.46 | <.001 | -10 | -30 | 6 |
|  |  |  |  |  |  | .077 | 3.31 | .001 | -6 | -14 | 10 |
|  | HC < SZ | .037 | 154 | .082 |  | .023 | 3.92 | <.001 | -24 | -24 | 12 |
|  |  |  |  |  |  | .155 | 3.13 | .001 | -14 | -34 | 8 |
| Right Motor | HC < SZ | .047 | 127 | .112 |  | .011 | 4.17 | <.001 | 18 | -24 | 8 |
|  |  | .239 | 12 | .635 |  | .210 | 2.95 | .002 | 6 | -24 | 6 |
|  | SIB < SZ | .106 | 61 | .264 |  | .068 | 3.59 | <.001 | 16 | -10 | 0 |
| Left Occipital | HC < SIB | .236 | 11 | .689 |  | .084 | 3.35 | .001 | -4 | -20 | 16 |
|  | SZ < SIB | .227 | 17 | .557 |  | .149 | 3.25 | .001 | -6 | -18 | 14 |
| Left Prefrontal | SZ < HC | .020 | 239 | .049 |  | .043 | 3.64 | <.001 | -6 | -32 | 2 |
|  |  |  |  |  |  | .049 | 3.58 | <.001 | -18 | -26 | 4 |
|  |  |  |  |  |  | .251 | 2.83 | .003 | -14 | -32 | -6 |
| Right Somatosensory | HC < SIB | .207 | 17 | .592 |  | .070 | 3.43 | .001 | 8 | -28 | -2 |
|  | HC < SZ | .176 | 23 | .577 |  | .014 | 3.98 | <.001 | 28 | -22 | -6 |
|  |  | .050 | 145 | .153 |  | .060 | 3.42 | .001 | 4 | -6 | -6 |
|  |  |  |  |  |  | .092 | 3.24 | .001 | 10 | -6 | 6 |
|  |  |  |  |  |  | .095 | 3.23 | .001 | 18 | -20 | 2 |
| Left Temporal | HC < SZ | .006 | 345 | .013 |  | .006 | 4.41 | <.001 | -24 | -24 | -4 |
|  |  |  |  |  |  | .044 | 3.69 | <.001 | -8 | -22 | 8 |
|  |  |  |  |  |  | .083 | 3.42 | .001 | -22 | -24 | 4 |
|  | SIB < SZ | .083 | 84 | .198 |  | .049 | 3.76 | <.001 | -24 | -26 | -2 |
|  |  |  |  |  |  | .225 | 3.00 | .002 | -10 | -30 | 6 |

Notes: results were significant at voxel-wise uncorrected *p* < .005 and cluster extent threshold ≥ 10 voxels.

FWE-corr, Family-wise error corrected; HC, healthy control participants; SIB, healthy siblings; SZ, persons with schizophrenia; uncorr, uncorrected.

**Table S5.** Average head rotations (absolute values) and displacements from both phase-encoding directions.

|  |  | SZ (*N* = 22) | | SIB (*N* = 20) | | HC (*N* = 44) | |  |  |
| --- | --- | --- | --- | --- | --- | --- | --- | --- | --- |
| Metrics | Direction | Mean | SD | Mean | SD | Mean | SD | *F* | *p* |
| Mean x rotation | PA | 0.23 | 0.23 | 0.20 | 0.21 | 0.18 | 0.25 | 0.41 | .67 |
|  | AP | 0.14 | 0.20 | 0.08 | 0.10 | 0.18 | 0.29 | 1.26 | .29 |
| Mean y rotation | PA | 0.12 | 0.10 | 0.11 | 0.11 | 0.11 | 0.12 | 0.09 | .91 |
|  | AP | 0.15 | 0.22 | 0.06 | 0.08 | 0.11 | 0.12 | 2.06 | .13 |
| Mean z rotation | PA | 0.14 | 0.13 | 0.08 | 0.10 | 0.10 | 0.08 | 2.47 | .09 |
|  | AP | 0.16 | 0.18 | 0.12 | 0.07 | 0.12 | 0.12 | 0.74 | .48 |
| Mean absolute total displacement | PA | 1.70 | 0.24 | 1.57 | 0.19 | 1.52 | 0.41 | 2.17 | .12 |
|  | AP | 1.59 | 0.48 | 1.34 | 0.16 | 1.39 | 0.36 | 2.91 | .06 |
| Mean relative total displacement | PA | 0.61 | 0.18 | 0.55 | 0.19 | 0.52 | 0.18 | 1.71 | .19 |
|  | AP | 0.57 | 0.18 | 0.55 | 0.22 | 0.50 | 0.16 | 1.59 | .21 |

Notes: AP, anterior-posterior phase-encoding direction; HC, healthy control participants; PA, posterior-anterior phase-encoding direction; SIB, healthy siblings; SZ, persons with schizophrenia.

**
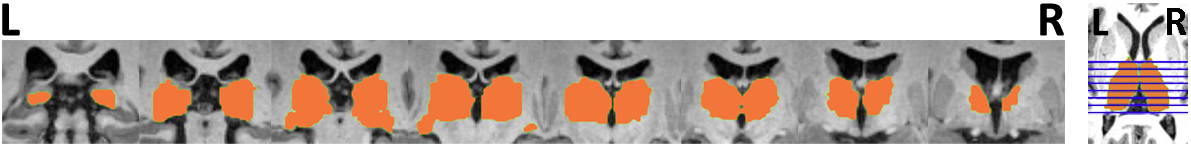
**

**Figure S1.** Thalamus mask (orange) for an example participant, displayed on the participant’s T1 image in coronal view on multiple slices from posterior to anterior.

**
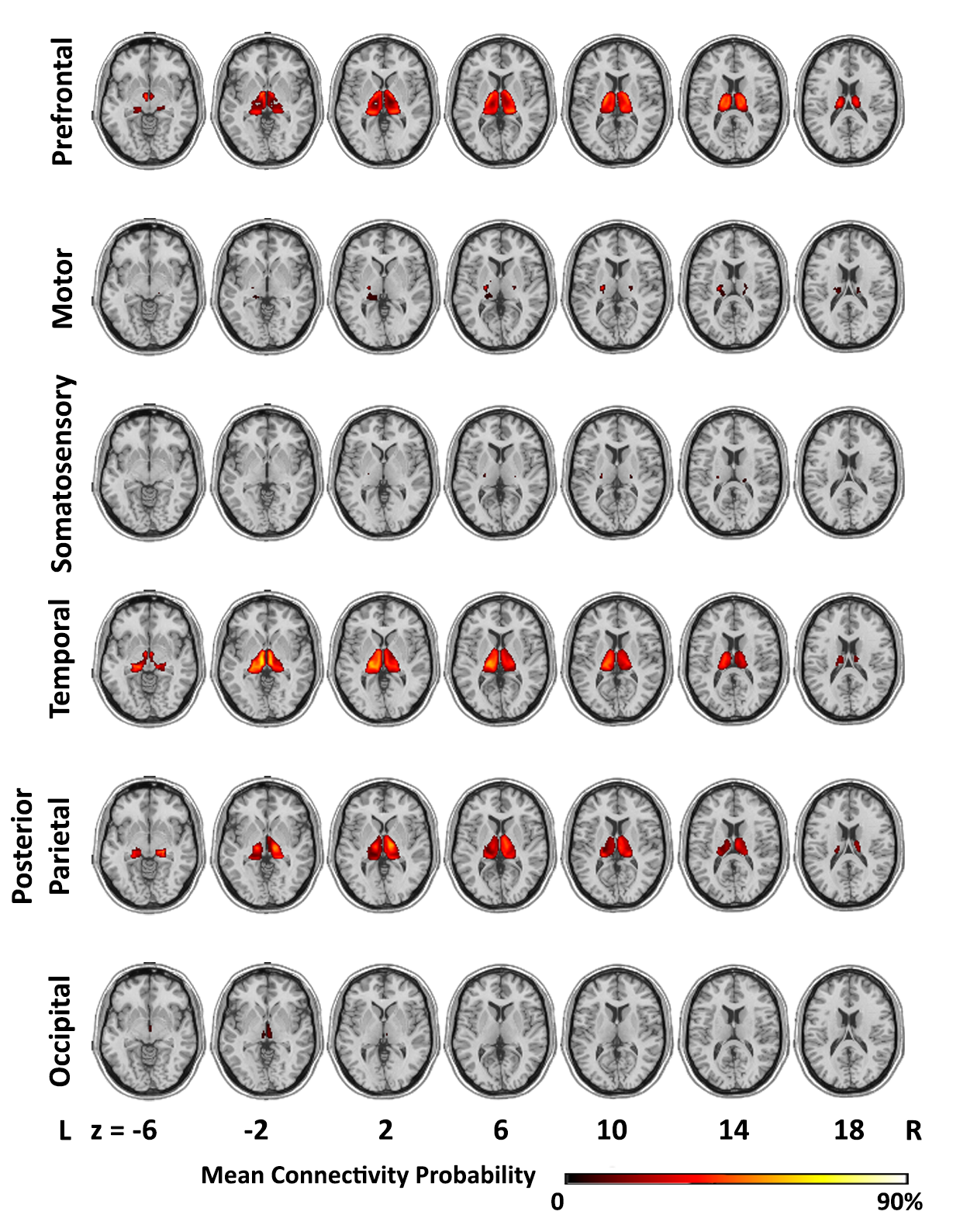
**

**Figure S2.** Thalamocortical connectivity voxel-wise probability maps across all groups in multi-slice axial view. Each row displayed voxel-wise probability maps indicating probability of connectivity of thalamus voxels with each cortical region of interest. For visualization purpose only, maps for motor, somatosensory, and occipital cortex were thresholded at 5%, and maps for prefrontal, temporal, and posterior parietal cortex were thresholded at 10%. Numbers in the bottom are the MNI coordinates.

**Supplementary References**

1. Andreasen NC, Flaum M, Arndt S (1992): The Comprehensive Assessment of Symptoms and History (CASH). An instrument for assessing diagnosis and psychopathology. *Arch Gen Psychiatry*. 49: 615–623.

2. Tabachnick BG, Fidell LS (2013): *Using Multivariate Statistics*, 6th ed. Pearson Education, Inc.

3. Roiser JP, Linden DE, Gorno-Tempini ML, Moran RJ, Dickerson BC, Grafton ST (2016): Minimum statistical standards for submissions to Neuroimage: Clinical. *NeuroImage Clin*. 12: 1045–1047.

4. Lieberman MD, Cunningham WA (2009): Type I and Type II error concerns in fMRI research: re-balancing the scale. *Soc Cogn Affect Neurosci*. 4: 423–428.

5. Steiger JH (1980): Tests for comparing elements of a correlation matrix. *Psychol Bull*. 87: 245–251.
